# Performance Comparison of Different Flat Plate Solar Collectors by Means of the Entropy Generation Rate Using Computational Fluid Dynamics

**DOI:** 10.3390/e25040621

**Published:** 2023-04-06

**Authors:** J. J. Ramírez-Minguela, V. H. Rangel-Hernández, J. A. Alfaro-Ayala, F. Elizalde-Blancas, B. Ruiz-Camacho, O. A. López-Núñez, C. E. Alvarado-Rodríguez

**Affiliations:** 1 Department of Chemical Engineering, Division of Natural and Exact Sciences, University of Guanajuato, Guanajuato 36050, Mexico; ja.alfaroayala@ugto.mx (J.A.A.-A.); beatriz.ruiz@ugto.mx (B.R.-C.); ce.alvarado@ugto.mx (C.E.A.-R.); 2 Department of Mechanical Engineering, Engineering Division, Campus Irapuato-Salamanca, University of Guanajuato, Salamanca 36885, Mexico; vrangel@ugto.mx (V.H.R.-H.); franciscoeb@ugto.mx (F.E.-B.); 3 Facultad de Ingeniería, Universidad Autónoma de Baja California, Mexicali 21280, Mexico; oscar.lopez.nunez@uabc.edu.mx

**Keywords:** CFD, zigzag geometries, conventional geometry, entropy generation analysis, FPCs

## Abstract

In this work, a numerical analysis of three different flat plate solar collectors was conducted using their entropy generation rates. Specifically, the Computational Fluid Dynamics (CFD) technique was used to compare the detailed performance of conventional and zigzag tube geometries of flat plate solar collectors (FPCs) in terms of their entropy generation rates. The effects of fluid viscosity, heat transfer, and heat loss of the flat plate solar collectors were considered for the local and global entropy generation rate analyses. Variations on the inlet volumetric flow rate of the FPCs from 1.0 to 9.0 L/min were simulated under the average solar radiation for one year in the state of Guanajuato, Mexico. The results illustrate and discuss the temperatures, pressures, and global entropy generation rates for volumetric flow variations. The velocity, temperature, and pressure distributions and the maps of the local entropy generation rates inside the collectors are presented and analyzed for the case with a flow rate of 3.0 L/min. These results demonstrate that the zigzag geometries achieved higher outlet temperatures and greater entropy generation rates than the conventional geometry for all the volumetric flow rates considered.

## 1. Introduction

The global production of energy from fossil fuels has been increasing considerably in recent years, resulting in an intensification of greenhouse gases. The use of renewable energies is necessary to diminish the effects of fossil fuels on the environment. In this sense, the use of the solar energy harnessed by water solar collectors is an alternative renewable energy source that can help to reduce the harmful effects of greenhouse gas production in the environment [1]. Several types of water solar collectors have been cited in the literature; however, flat plate solar collectors (FPCs) and evacuated tube solar water collectors (ETCs) are typically used for domestic applications [2]. An ETC is constructed by joining several tubes to a header pipe, with the tubes being evacuated to minimize heat transfer loss to the environment. A FPC is constructed by two horizontal headers pipes, one at the top of the FPC and the other at the bottom, with several vertical pipes connected in parallel between them. Some FPCs are constructed with fins inside and/or outside the vertical parallel tubes, and their header and vertical parallel pipes are protected by a transparent cover (commonly tempered glass) to improve heat transfer [3]. Their simple construction, low price, and durability are some advantages that FPCs have over ETCs [4]. Although ETCs and FPCs commonly work with water, recent investigations have explored the use of nanofluids as working fluids [5,6].

Various experimental and numerical studies on the performance of flat plate solar collectors can be found in the literature [7,8,9,10]. Azad [11] conducted an experimental analysis, taking into consideration real operating conditions for the FPC, with different numbers of heat pipes in the two heat pipe solar collectors and varying the heat exchanger area. This researcher concluded that the efficiency of heat pipe solar collectors increases as the number of heat pipes increases, and the incoming energy in the heat pipe solar collector increases as the effective absorber area increases. Alwan et al. [12] designed and constructed a solar water distiller prototype with the help of a FPC. They experimentally evaluated the thermal performance of the FPC for several months in 2019, considering the weather conditions in Ekaterinburg, Russia. They showed that the maximum water temperature was obtained for higher solar radiation intensity and that the highest efficiency of the solar collector was recorded at midday with the highest solar energy intensity. Hashim et al. [13] designed a flat plate collector in a lope square pattern and studied its performance experimentally in the conditions of Baghdad, Iraq, using water as a fluid at two different flow rates: 5.3 and 6.51 L/min. They concluded that the system was useful because of the collector’s effectiveness and the high temperatures reached. Wei et al. [14] conducted an experimental and numerical analysis of a FPC with an integrated heat pipe. They developed a transient heat transfer model based on the energy balance for each component of the collector. The model validation was done using experimental data. They concluded that the modified collector could be used in the solar water heater system due to the convenient thermal performance reached. Kargarsharifabad et al. [15] analyzed the implementation of FPCs considering heat pipes in a building to provide its energy needs. They simulated a thermodynamic analysis of the system and performed a comparison between a pulsating heat pipe with FPCs and conventional solar collectors, considering exergy efficiency. Low exergy efficiency was found in the conventional collectors; however, for heat transfer between the reservoir and the absorption plate, the pulsating heat pipe with a FPC was preferred. Mansour [16] investigated the thermal performance of a new design of a minichannel-based solar flat plate collector. His research was based on a three-dimensional numerical model. The minichannel solar collector model was analyzed to calculate the heat and laminar fluid flow of water throughout the solar collector. The thermal performance of the proposed flat plate collector was compared with a conventional ETC, with a ETC with an evacuated heat-pipe tube, and with a conventional FPC. The results showed a better thermal performance considering the proposed design at high water flow rates; however, a worse hydraulic performance was obtained. Robles et al. [17] experimentally analyzed the performance of an aluminum-based minichannel solar heater under different climatic conditions throughout a year at the University of California, Merced. They analyzed the thermal performance of the proposed design and compared it with a conventional copper FPC considering the same climatic conditions. They also developed a mathematical model of the collector’s proposed design, and it was validated by means of their experimental data. Their results showed an improvement in the thermal efficiency of the collector. Deng et al. [18] experimentally studied the thermal performance of a novel FPC with a micro-channel heat pipe array (MHPA). Their results showed that the FPC-MHPA had an excellent thermal performance and a high efficiency. Azad E. [19] experimentally compared the thermal performance of three different types of heat pipe solar collectors at the same working conditions. The study concluded that the performances of the three types were satisfactory, each with its own advantages and disadvantages. Dovic and Andrassy [20] conducted numerical investigations on the performance of FPCs in order to find ways to improve their thermal efficiency by developing different numerical models. They analyzed the influence of several parameters on thermal efficiency, including tube diameter, absorber plate to glass cover distance, optical properties, etc. The study also explored possible replacements for the flat absorber surfaces commonly used in FPCs, including corrugated absorber surfaces. Finally, a new design for collectors consisting of chevron plates commonly used in plate heat exchangers was studied. García et al. [21] presented a comparative experimental analysis of the thermal performance of a FPC by considering three twisted tapes and three wire coil specimens inserted into a solar collector tube. They reported and discussed that the heat transfer, fluid friction, and fluid flow were related. Gunjo et al. [22] developed a CFD numerical model for a FPC to predict the thermal performance of a single novel bent tube collector attached to an absorber plate using exergy and energy analysis. Their results showed a higher performance using the novel geometry in comparison to the results that can be found in the literature. They also analyzed the impact of different climatic conditions, working fluids, inlet water temperatures, mass flow rates, and other factors on the FPC’s performance, showing that these parameters cause variations in the performance of the FPC.

The use of the second law of thermodynamics to evaluate the performance of flat plate solar collectors is scarce. Entropy generation is a measure of the degradation in the performance of flat plate solar collectors, and it depends greatly on irreversibilities presented during the process. The thermodynamic irreversibilities are adequately quantified by means of the entropy generation rate. In this sense, an efficient energy conversion device has a low entropy generation. Alim et al. [23] analyzed a conventional flat plate solar collector theoretically by means of entropy generation using different types of metal oxide nanofluids. The authors found that the CuO nanofluid reduced the entropy generation and enhanced the heat transfer coefficient compared to using water as an absorbing fluid. Finally, they concluded that the performance of a solar collector can be enhanced by using nanofluids instead of just water as an absorbing fluid. Jilani and Thomas [24] numerically studied the effects of the performance parameters of sheet and tube type solar flat plate collectors by means of the entropy generation rate. Their results were presented and discussed over a wide range of values for the aspect ratio of the absorber plate, overall loss parameter, and dimensionless fluid outlet temperature. They concluded that the assumption of constant thermal conductivity and overall loss coefficient results in overestimations of the total entropy generation rate with a substantial error. They also concluded that the total entropy generation rate increases with an increase in the overall loss parameter. Jouybari et al. [25] experimentally examined the effects of metal foams as passive thermal developers on the performance of water-based flat plate solar collectors. An entropy generation analysis was performed for two similar wetted absorber collectors, the first was a fully filled porous channel, and the second was an empty channel. The entropy generation analysis revealed that the portion of heat transfer irreversibility was more dominant in both collectors. Alklaibi et al. [26] experimentally investigated the performance of a flat plate solar collector using a nanodiamond nanofluid that circulated through the collector under thermosyphon conditions, considering different solar radiations. They analyzed the power consumption requirement, entropy generation, thermal efficiency, heat transfer, exergy efficiency, and friction factor within a time interval. Their outcomes showed that the analyzed parameters were highly influenced by changes in solar radiation.

Most of above studies reported on the performance of different geometries of flat plate solar collectors in terms of thermal efficiency. Concerning to the entropy generation rates analysis, they considered black box models. Therefore, this work focused on comparing the thermos-hydraulic performances of three different geometries of flat plate solar collectors and showed the irreversibilities inside of these devices by means of an entropy generation rate analysis. This study considered three different geometries of the flat plate solar collectors: (1) conventional, (2) zigzag type A, and (3) zigzag type B. The numerical simulations were performed considering a complete three-dimensional geometry with the help of CFD (ANSYS-FLUENT^®^). The velocities, pressures, and temperature contours of the three geometries were compared under the average climatic conditions that prevail in the state of Guanajuato, Mexico. Finally, the global and local entropy generation rates due to fluid viscosity, heat losses, and heat transfer were discussed.

## 2. Geometries and Mathematical Model

### 2.1. Configurations of the Geometries of the Solar Collectors

**Figure 1:**
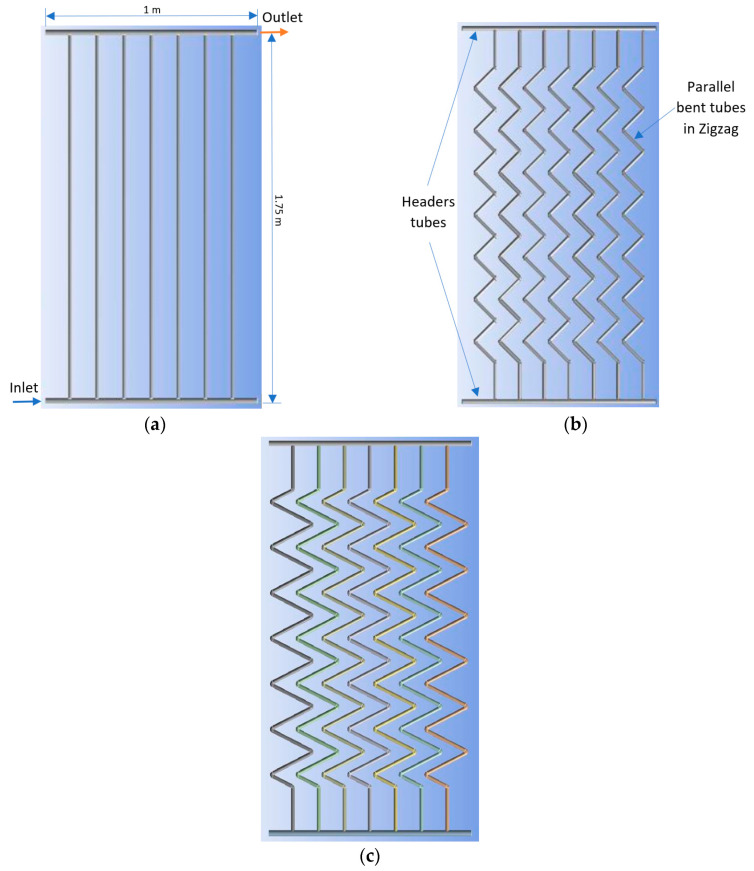
FPC geometries analyzed: (**a**) conventional, (**b**) zigzag type A, and (**c**) zigzag type B.

Three different flat plate solar collector geometries were analyzed:

1. Conventional: Consists of 7 parallel cooper and header tubes, Figure 1a.
2. Zigzag type A: Consists of 7 parallel bent and headers tubes, Figure 1b.
3. Zigzag type B: Consists of 7 parallel modified bent and header tubes, Figure 1c.

The geometrical parameters of the three flat plate solar collectors and the water properties used for the model are described in Table 1.

### 2.2. Equations Used in the Model

The equations used in the model are described by means of different partial differential equations that help to describe the physical phenomena inside the FPCs. To achieve a reduction in the computational cost and time taken for each case to reach convergence, certain assumptions were considered, including: the fluid was considered a Newtonian fluid, steady-state conditions were established, incompressible flow was considered, density change was considered according to the Boussinesq approximation (Equation (7)), and the other properties were considered constant.

The velocity and pressure distributions inside the FPCs were obtained by incorporating the laws of mass and momentum conservations in the model. These are described by the following partial differential equations:

(1) $$\nabla \cdot \rho \vec{V}=0$$

(2) $$\rho \left(\vec{V}\cdot \nabla \right)\vec{V}=-\nabla p+\mu \nabla^{2}\vec{V}+\vec{F}$$

where ρ is density, *V* is velocity, *μ* is dynamic viscosity, *p* is pressure, and *F* is the sum of the body forces due to gravity in the *y*-direction where the Boussinesq approximation is considered.

Moreover, because the variation of the inlet volumetric flow rate varies, laminar and turbulent flows were considered in this work. Consequently, the standard *k-ε* turbulence model was applied to solve the cases with turbulent flow, which can be expressed as follows [27]:

(3) $$\frac{\partial }{\partial x_{i}}\left(\rho ku_{i}\right)=\frac{\partial }{\partial x_{i}}\left[\left(\mu +\frac{\mu_{t}}{\sigma_{k}}\right)\frac{\partial k}{\partial x_{j}}\right]+G_{k}+G_{b}-\rho \varepsilon -Y_{M}$$

(4) $$\frac{\partial }{\partial x_{i}}\left(\rho \varepsilon u_{i}\right)=\frac{\partial }{\partial x_{i}}\left[\left(\mu +\frac{\mu_{t}}{\sigma_{\varepsilon }}\right)\frac{\partial \varepsilon }{\partial x_{j}}\right]+C_{1\varepsilon }\frac{\varepsilon }{k}\left(G_{k}\right)-C_{2\varepsilon }\rho \frac{\epsilon^{2}}{k}$$

where $G_{k}$ is the generation of turbulence kinetic energy due to the mean velocity gradients, $G_{b}$ represents the generation of turbulence kinetic energy due to buoyancy effects, $Y_{M}$ is the contribution of the fluctuating dilatation in compressible turbulence to the overall dissipation rate, $C_{1\varepsilon }$ and $C_{2\varepsilon }$ are constants, and $\mu_{t}$ is the turbulent viscosity which is calculated as follows:

(5) $$\mu_{t}=\rho C_{\mu }\frac{k^{2}}{\varepsilon }$$

The temperature distribution inside the FPCs was obtained by solving the energy equation, which is defined by the following partial differential equation:

(6) $$\rho c\left(u_{x}\frac{\partial T}{\partial x}+u_{y}\frac{\partial T}{\partial y}+u_{z}\frac{\partial T}{\partial z}\right)=k\left(\frac{\partial^{2}T}{\partial x^{2}}+\frac{\partial^{2}T}{\partial y^{2}}+\frac{\partial^{2}T}{\partial z^{2}}\right)$$

where c is the specific heat, $u_{x}$, $u_{y}$, and $u_{z}$ are the velocity components, k is the conductivity, and *T* is the temperature. The buoyancy phenomenon was considered by means of the Boussinesq approximation (BA) as follows:

(7) $$\rho_{\infty }-\rho =\rho \beta \left(T-T_{\infty }\right)$$

where *β* is the volumetric thermal expansion coefficient.

### 2.3. Computing the Irreversibilities Using the Entropy Generation Rate

The local entropy generation rate is calculated as:

(8) $$\begin{array}{ll} s_{i} & =\frac{k}{T^{2}}\left[{\left(\frac{\partial T}{\partial x}\right)}^{2}+{\left(\frac{\partial T}{\partial y}\right)}^{2}+{\left(\frac{\partial T}{\partial z}\right)}^{2}\right]\\ & +\frac{\mu }{T}\left\{2\left[{\left(\frac{\partial u_{x}}{\partial x}\right)}^{2}+{\left(\frac{\partial u_{y}}{\partial y}\right)}^{2}+{\left(\frac{\partial u_{z}}{\partial z}\right)}^{2}\right]+{\left(\frac{\partial u_{x}}{\partial y}+\frac{\partial u_{y}}{\partial x}\right)}^{2}+{\left(\frac{\partial u_{x}}{\partial z}+\frac{\partial u_{z}}{\partial x}\right)}^{2}+{\left(\frac{\partial u_{y}}{\partial z}+\frac{\partial u_{z}}{\partial y}\right)}^{2}\right\} \end{array}$$

As can be noted in Equation (8), the local entropy generation rate is due to heat transfer, $s_{h}$ and viscous stress, $s_{µ}$*:*

(9) $$s_{i}=s_{h}+s_{µ}$$

The global entropy generation rate can be calculated as:

(10) $$S_{i}=\int s_{i}dV$$

The entropy generation rate due to heat loss, $S_{q}$, is considered as:

(11) $$S_{q}=\frac{{\dot{Q}}_{loss}}{T_{env}}$$

where $T_{env}$ is the temperature of the environment and ${\dot{Q}}_{loss}$ is the heat loss which can be calculated as follows:

(12) $${\dot{Q}}_{loss}={\dot{Q}}_{sun,in}-{\dot{Q}}_{useful}$$

where ${\dot{Q}}_{sun,in}$ is the solar flux and ${\dot{Q}}_{useful}$ is the useful heat. The ${\dot{Q}}_{sun,in}$ is calculated as in [28]:

(13) $${\dot{Q}}_{sun,in}=A_{c}I_{T}$$

where $A_{c}$ is the absorber area of the solar collector and $I_{T}$ is the incident solar energy per unit area. The ${\dot{Q}}_{useful}$ was calculated as follows [12,29,30]:

(14) $${\dot{Q}}_{useful}=\dot{m}\cdot c\cdot \left(T_{out}-T_{in}\right)$$

where $\dot{m}$ is the mass flow rate, $T_{out}$ is the outlet temperature of the FPC, and the $T_{in}$ is the inlet temperature of the FPC. Therefore, two contributions of the entropy generation ($S_{i}$ and $S_{q}$) are needed to calculate the total entropy generation:

(15) $$S_{total}=S_{i}+S_{q}$$

### 2.4. Boundary Conditions of the Model

A range of volumetric flow rates from 1.0 to 9.0 L/min at the inlet of the FPC was considered. The average solar radiation, 9.4 kWh/m^2^∙day, for one year in the state of Guanajuato, Mexico, was used. A pressure outlet boundary condition was applied at the exit of the FPC, and a heat flux (${\dot{Q}}_{useful}$) in the walls of the FPC was defined. The useful heat was obtained analytically by subtracting the optical and thermal losses in the tubes and the headers from the total amount of heat received due to solar radiation, as presented by Li et al. [29] and Budihardjo et al. [30]. Experimentally, the useful heat is the heat required for increasing the temperature of the water between the entrance and the exit (Equation (14)). All these boundary conditions were considered in the CFD numerical model in order to simulate the effect of solar radiation and predict the thermal and hydraulic performance of the flat plate solar collectors.

### 2.5. Numerical Approach

**Figure 2:**
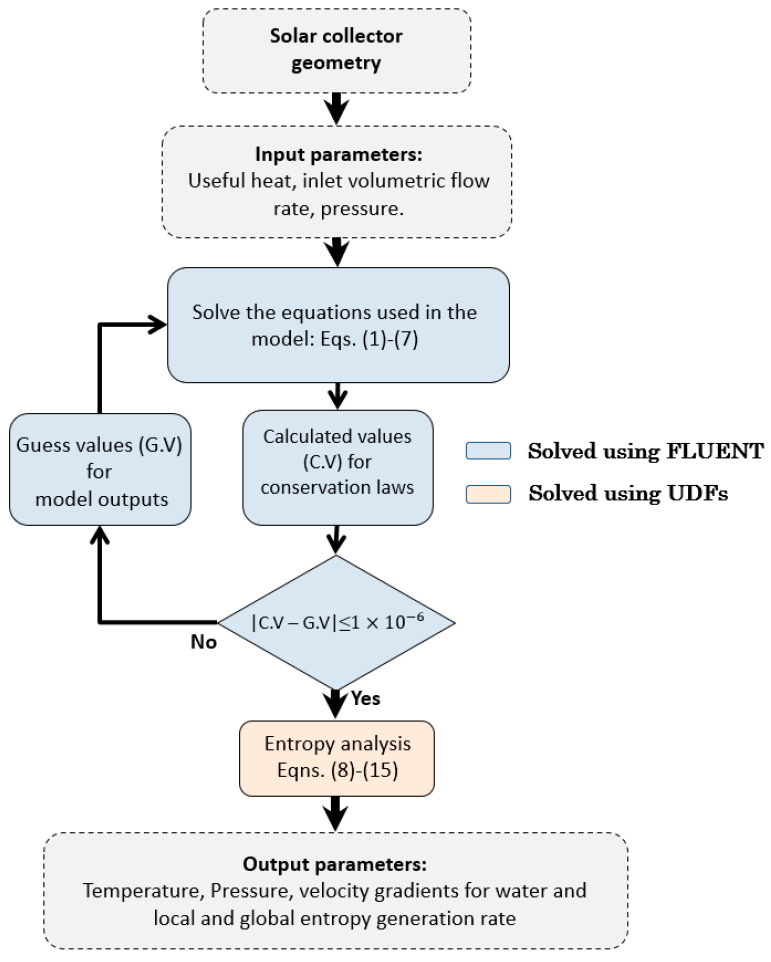
Model algorithm for simulating the FPCs.

An iterative algorithm was implemented with the help of the ANSYS Fluent^®^ v.18.1 software to solve the governing equations described in Section 2.2 and Section 2.3 (see Figure 2). The SIMPLE algorithm was used to solve Equations (1)–(7) and to obtain the velocity and temperature fields inside the FPC. The convergence criterion was considered once the residuals were lower than 10^−6^. Finally, the entropy generation analysis was computed through user-defined functions (UDFs) (Equations (8)–(15)).

A sensibility study of the mesh was carried out. Therefore, the computational grid was subsequently refined for case 1 from 30,253 to 1,734,153 cells, for case 2 from 516,833 to 2,501,458 cells, and for case 3 from 405,458 to 4,589,410 cells. The effects on the average outlet temperature of the FPCs at a volumetric flow rate of 3 L/min was monitored. The results showed that a structured mesh of 649,887, 1,260,857, and 1,923,992 elements were sufficient to achieve mesh-independent solutions for case 1 (conventional), case 2 (zigzag type A), and case 3 (zigzag type B), respectively (Table 2, Table 3 and Table 4).

**Figure 3:**
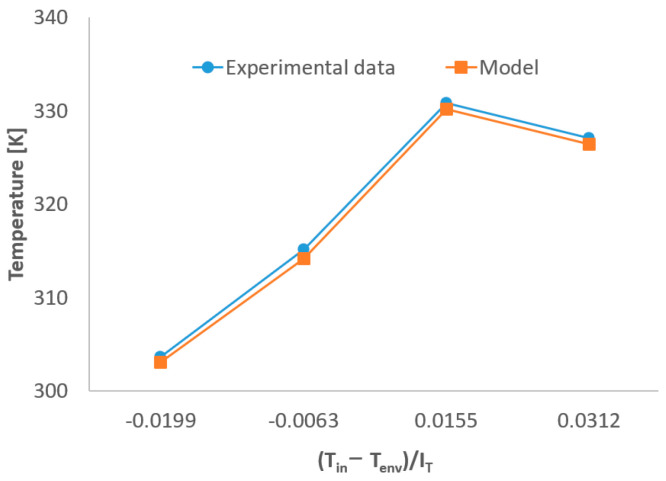
Comparison of the average temperature at the outlet of the flat plate solar collector: model developed and experimental data [31].

Moreover, the research of Zambolin et al. [31] was considered to validate the model of the FPC. They carried out an experimental analysis of the thermal performance of a FPC under stationary, standard, and daily conditions at the University of Padova, Padova, Italy. The experimental operating conditions and the geometrical parameters were replicated by applying the previously described equations to the model, and the results obtained were reliable (Figure 3).

## 3. Results

### 3.1. Thermal Performance Comparison of the FPCs: Variable Volumetric Flow Rates

**Figure 4:**
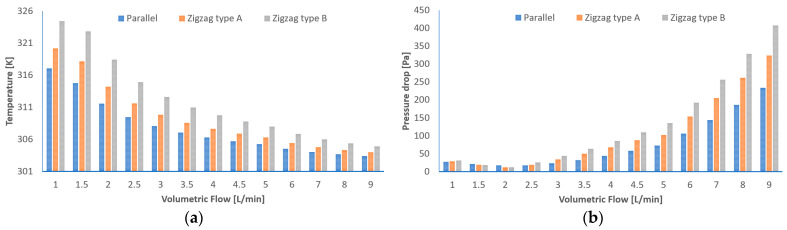
Performance comparisons at different volumetric flows: (**a**) temperature outlet, and (**b**) pressure drop.

Figure 4 shows a comparison of the outlet temperature and pressure drop between the conventional and zigzag tube geometries of flat plate solar collectors at varying volumetric flow rates (ranging from 1.0 L/min to 9.0 L/min) and a constant horizontal radiation of 9.4 kWh/m^2^∙day. As depicted, the outlet temperatures obtained in the zigzag type B (case 3) geometry were higher than those in the conventional (case 1) and zigzag type A (case 2) geometries. The highest temperature of the water at the outlet of the solar collector, approximately 324.5 K, was achieved in the zigzag type B geometry at a volumetric flow rate of 1.0 L/min, while the lowest temperature of the water at the outlet of the solar collector, approximately 303 K, was achieved in the conventional case (case 1) at a volumetric flow rate of 9.0 L/min. Moreover, the maximum temperature differences were reached at 1.0 L/min, where the temperature difference between case 3 and case 1 was approximately 7.4 K, and the temperature difference between case 3 and case 2 was approximately 3.1 K. The minimum temperature differences were reached at 9.0 L/min, where the temperature difference between case 3 and case 1 was approximately 1.5 K, and between case 3 and case 2, the temperature difference was approximately 0.6 K (Figure 4a). It is important to highlight that this behavior was mainly due to the differences in the area of the walls of the parallel tubes. Case 3 had a larger wall area than conventional case 1, which was 62% greater than case 1 and 31% greater than case 2. Despite this, the three geometries occupied the same area of 1.75 m^2^, as shown in Figure 1.

The pressure drop behavior at different volumetric flow rates is illustrated in Figure 4b. As can be seen, the pressure drop between the three geometries was almost the same for volumetric flow rates lower that 2.5 L/min. In this sense, from 1.0 to 2.0 L/min, the pressure tended to decrease slightly due to the higher temperature reached at these volumetric flow rates, which provoked considerable density changes in the water inside the FPCs. Furthermore, the pressure inside the flat plate solar collectors tended to increase in all three cases as the volumetric flow rates increased after 2.5 L/min. The pressure drop at volumetric flow rates of 2.5, 5.0, and 9.0 L/min for the zigzag type B geometry was approximately 25.8, 135, and 408.3 Pa, respectively. For the zigzag type A geometry, the pressure drop was approximately 18.9, 102.4, and 324.1 Pa, respectively, and for the conventional geometry, it was approximately 17.4, 72.9, and 234 Pa, respectively. It is noteworthy that the conventional geometry had a lower pressure drop than the zigzag geometries at the different volumetric flow rates. The minimum and the maximum pressure reached in the FPCs were reached at 2.0 L/min and 9.0 L/min, respectively. The biggest difference in the maximum pressure between case 3 and case 1 at 9.0 L/min was approximately 75%, as shown in Figure 4b. This was due to the zigzag effect in fluid flow that caused higher pressure gradients than the conventional geometry.

### 3.2. Performance Comparison of the FPCs at 3.0 L/min

**Figure 5:**
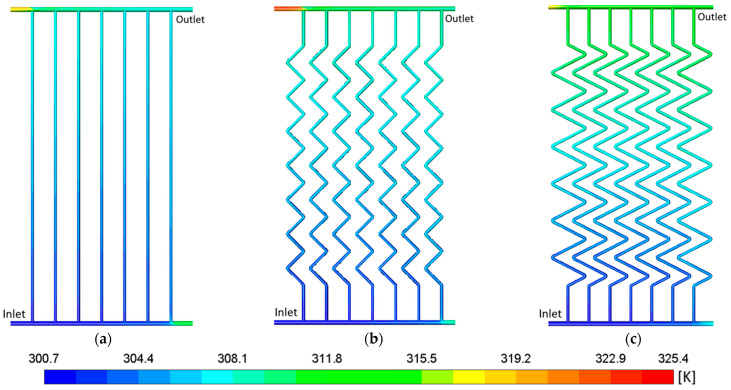
Temperature distribution inside the FPCs at 3.0 L/min: (**a**) conventional, (**b**) zigzag type A, and (**c**) zigzag type B.

**Figure 6:**
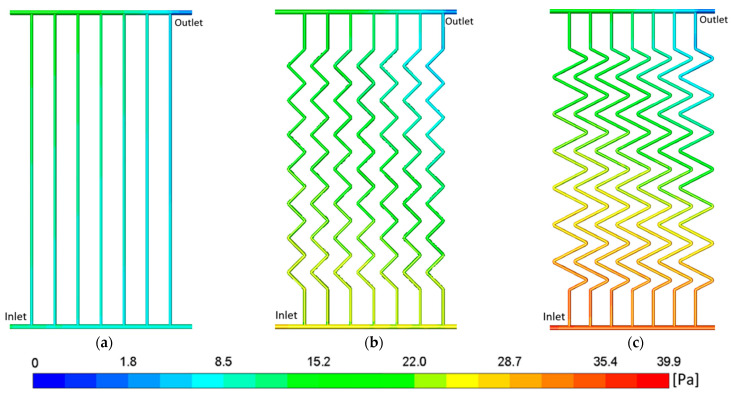
Pressure distribution inside the FPCs at 3.0 L/min: (**a**) conventional, (**b**) zigzag type A, and (**c**) zigzag type B.

**Figure 7:**
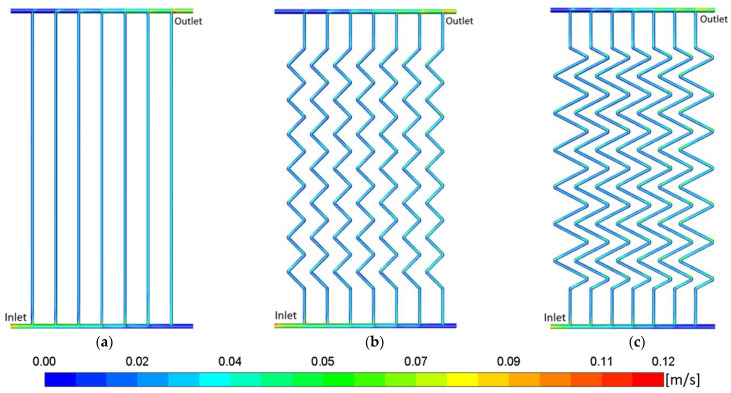
Velocity distribution inside the FPCs at 3.0 L/min: (**a**) conventional, (**b**) zigzag type A, and (**c**) zigzag type B.

A detailed comparison of the fields of temperature, pressure, and velocity inside the pipes that made up the FPCs at a volumetric flow rate of 3.0 L/min are shown in Figure 5, Figure 6 and Figure 7, respectively. This flow rate was selected because, at lower flow rates, density changes according to the Boussinesq approximation become more significant, which would affect the pressure drop in the FPCs. Consequently, a similar trend in the pressure drops of the three FPCs could be seen as the volumetric flow rate increased (Figure 4b). Figure 5 shows that the temperature increased from the lower header towards the upper header for all three of the FPC geometries. The highest temperatures were reached in the upper header opposite to the outlet flow for all three FPCs. It was observed that in the zigzag type A geometry, a maximum temperature of approximately 325 K was reached, while in the conventional geometry, a maximum temperature of approximately 321 K was reached. Moreover, the highest temperatures in the lower header were reached in the opposite side of the inlet flow for all three geometries. As can be noted in Figure 4a, the outlet temperature of the conventional geometry, the zigzag type A, and the zigzag type B at a flow rate of 3.0 L/min were approximately 308 K, 310 K, and 312.6 K, respectively. 

The pressure in the interior of the pipes of the conventional (case 1), zigzag type A (case 2), and zigzag type B (case 3) configurations of the FPCs at 3.0 L/min is shown in Figure 6. The maximum pressure gradient inside the tubes of the conventional configuration was approximately 20 Pa, for the zigzag type A configuration was approximately 30 Pa, and for the zigzag type B was approximately 40 Pa. As can be observed, the lowest and highest pressure drops were achieved in cases 1 and 3, respectively. The increase in pressure drop in the zigzag geometries was due to the several deviations in each tube that the fluid flow experienced on its way to the outlet, from the lower header to the upper header, and due to the use of longer tubes in the construction of the zigzag geometries compared to the conventional geometry. The longest tubes corresponded to case 3, which had the highest pressure drop, and the shortest tubes corresponded to case 1, which had the lowest pressure drop. Finally, it can be observed that the pressure field through the pipes of the conventional configuration of the collector was more homogeneous than the pressure field in the cases with zigzag configurations.

Figure 7 illustrates the velocity field in the interior of the pipes of the conventional (case 1), zigzag type A (case 2), and zigzag type B (case 3) configurations of the FPCs for a volumetric flow rate of 3.0 L/min. The velocity field for all three cases was almost the same due to the same diameters of the headers and the tubes considered (Figure 7). Near the inlet and outlet of the headers of the solar collector, the velocity of the water was approximately 0.08 m/s. The fluctuations in velocity in the interior of the pipes that make up the FPCs were mainly due to the seven divisions that fluid underwent as it flowed from the lower header to the upper header. The lowest velocities were obtained in the corners opposite to the inlet and outlet of the fluid flow in the FPCs, (Figure 7). In these sections, the movement of the fluid was mainly influenced by the temperature effect on the density of the water, which was approximated using the Boussinesq equation (Equation 7). In this sense, the highest temperatures reached inside the FPCs (Figure 5) were mainly due to the low velocities that the FPCs had in these zones (see Figure 7).

### 3.3. Entropy Generation Analysis

**Figure 8:**
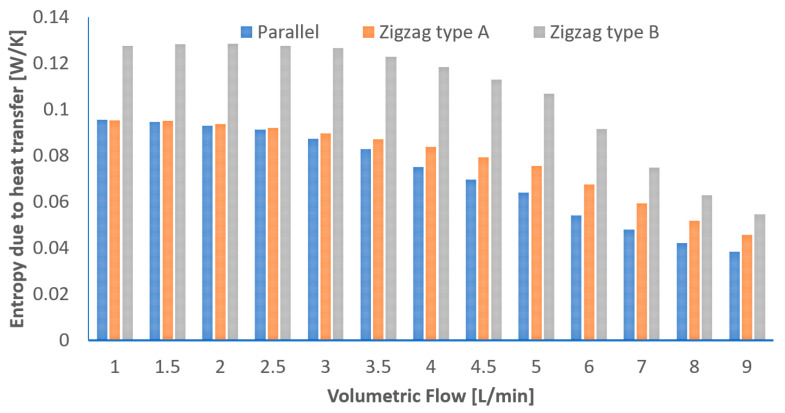
Entropy generation rate due to heat transfer, *S_h_*.

Figure 8 shows the entropy generation rate due to heat transfer (*S_h_*), fluid viscosity (*S_µ_*), and heat loss (*S_q_*), and the global entropy generation rates (*S_total_*) at different volumetric flow rates ranging from 1.0 to 9.0 L/min for the three cases. In general, for all three cases, *S_h_* decreased as the volumetric flow rate increased. For example, at volumetric flow rates of 1.0, 5.0, and 9.0 L/min, the conventional geometry had *S_h_* values of approximately 0.0955, 0.0639, and 0.0384 W/K, respectively, while the zigzag type A had *S_h_* values of approximately 0.0954, 0.0755 and 0.0457 W/K, respectively, and the zigzag type B had *S_h_* values of approximately 0.1276, 0.1070 and 0.0545 W/K, respectively. These findings indicate that the temperature gradients inside the FPC tubes decreased when the volumetric flow rates increased. As can be observed, the *S_h_* values between cases 1 and 2 were almost the same, 0.095 W/K, for volumetric flow rates lower than 2.5 L/min, while case 3 had an increase of approximately 40% at the same volumetric flow rates. Moreover, as the volumetric flow rate increased from 2.5 to 9.0 L/min, the entropy generation due to heat transfer was higher in case 2 compared to the case 1, up to 25%. Although the trends in the entropy generation decreased in all cases as the volumetric flow rate increased, the maximum difference in the entropy generation between case 1 and case 3 was approximately 70%, and between case 3 and 2 was approximately 42%. Furthermore, the entropy generation rate due to heat transfer for cases 1, 2, and 3 diminished to 60%, 52%, 57%, respectively, for the lowest volumetric flow rate (1.0 L/min) and the highest volumetric flow rate (9.0 L/min).

**Figure 9:**
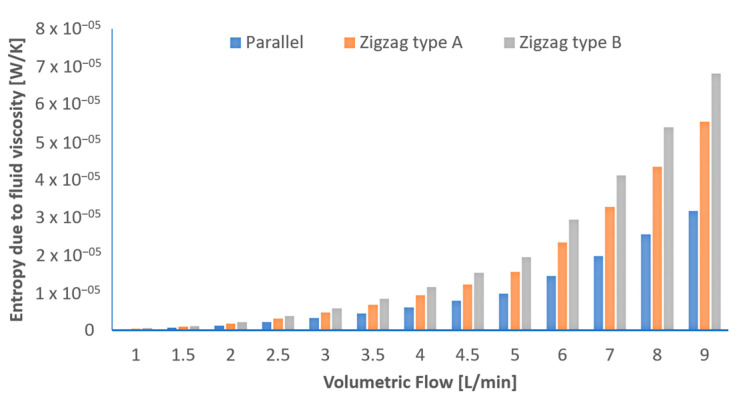
Entropy generation rate due to fluid viscosity, *S_µ_*.

The entropy generation rate due to the fluid viscosity, *S_µ_*, is shown in Figure 9. As was expected, the entropy due to fluid viscosity increased as the volumetric flow rate increased. Figure 9 shows that the lowest and highest entropy generation rates for the FPC in all the cases were reached at 1.0 L/min and 9.0 L/min, respectively. The maximum differences in the entropy generation rate due to the fluid viscosity between case 3 and case 1 were more than twice the value of case 1 at 9.0 L/min. This variation between the conventional and the zigzag type B geometries was due to the zigzag effect in fluid flow. In other words, the fluid flow through the parallel tubes from the lower header to the upper header mostly flowed in one direction, whereas the fluid flow through the zigzag geometry experienced several deviations to arrive at the upper header from the lower header. Hence, the difference in *S_µ_* between case 3 and case 2 at 9.0 L/min was smaller, approximately 23%, as both geometries had the same zigzag effect. Case 3 had the highest *S_µ_* value because its geometry had longer tubes compared to case 2.

**Figure 10:**
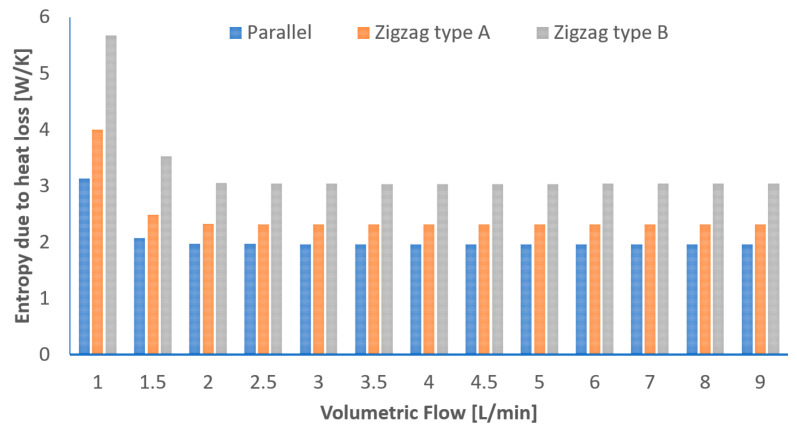
Entropy generation rate due to heat loss, *S_q_*.

The comparison of the entropy generation rate due to heat loss, *S_q_*, for the conventional (case 1), zigzag type A (case 2), and zigzag type B (case 3) configurations of the FPCs, considering volumetric flow rates ranging from 1.0 L/min to 9.0 L/min, is illustrated in Figure 10. As can be observed, there was a decrease in the values of *S_q_* for all three cases at volumetric flow rates of 1.0 L/min to 9 L/min. For example, in case 1, the *S_q_* values for volumetric flow rates of 1, 2, 3, and 5 L/min were 3.1334, 1.9730, 1.9634 and 1.9588 W/K, respectively. In case 2, the *S_q_* values for volumetric flow rates of 1, 2, 3, and 5 L/min were 3.9952, 2.3223, 2.3130 and 2.3127 W/K, respectively. Similarly, in case 3, the *S_q_* values for volumetric flow rates of 1, 2, 3, and 5 L/min were 5.6719, 3.04851, 3.0375 and 3.0334 W/K, respectively. It is worth mentioning that the entropy generation rate due to heat loss was mainly related to the absorptivity and transmittivity of the material used in the collectors, along with the weather conditions such as ambient temperature, wind velocity, direct radiation, diffuse radiation, and total radiation. For all the volumetric flow rates, the highest *S_q_* values were related to case 3, and the lowest values were related to case 1. The maximum difference between case 3 and case 1 was approximately 81%, and for case 3 and 2 it was approximately 42%, and both were observed at 1.0 L/min. These results were due to differences in the area of the walls of the parallel tubes, as was discussed in Section 3.1.

**Figure 11:**
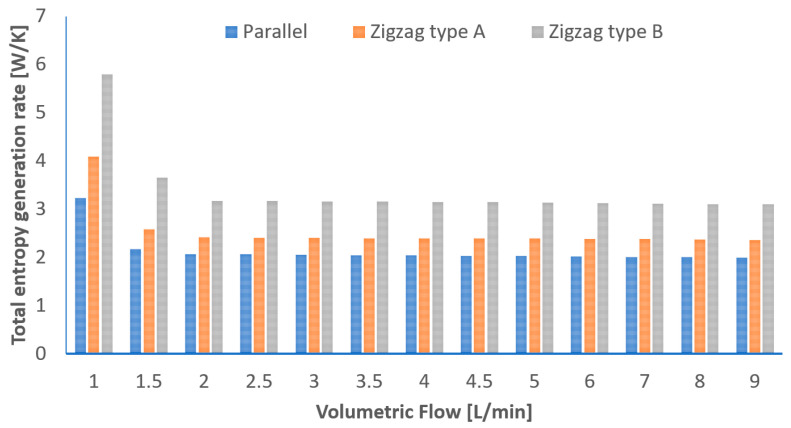
Total entropy generation rate, *S_total_*.

Figure 11 illustrates a comparison of the total entropy generation rate, *S_total_*, for the conventional (case 1), zigzag type A (case 2), and zigzag type B (case 3) configurations of the FPCs considering volumetric flow rates of 1.0 L/min to 9.0 L/min. As expected, the *S_total_* exhibited the same trend as the *S_q_* (Figure 10). This behavior was due to the significant contribution of the entropy generation rate due to the heat loss associated with the construction materials (pipes, headers, type of cover) of the FPCs and the related phenomena such as optical and heat transfer losses by solar radiation.

Finally, based on the analysis in Figure 8, Figure 9, Figure 10 and Figure 11, it can be observed that the values obtained for *S_µ_* were smaller in comparison to the entropy generation due to heat transfer and heat loss. Therefore, it can be established that for the operating conditions considered for these three geometries, *S_µ_* was negligible. It can also be observed that the maximum contribution of the *S_h_* to the *S_total_* was approximately 4.2% for the zigzag type B geometry with a volumetric flow rate of 3.0 L/min.

### 3.4. Maps of the Local Entropy Generation Rates inside the Tubes of the FPCs at 3.0 L/min

**Figure 12:**
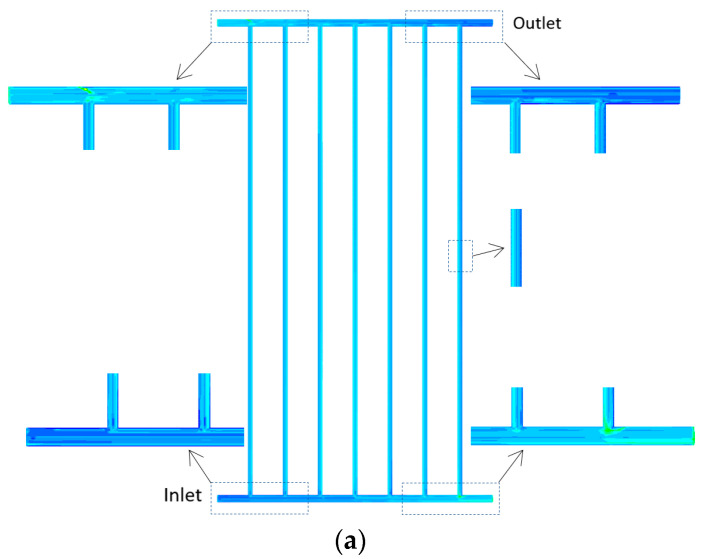

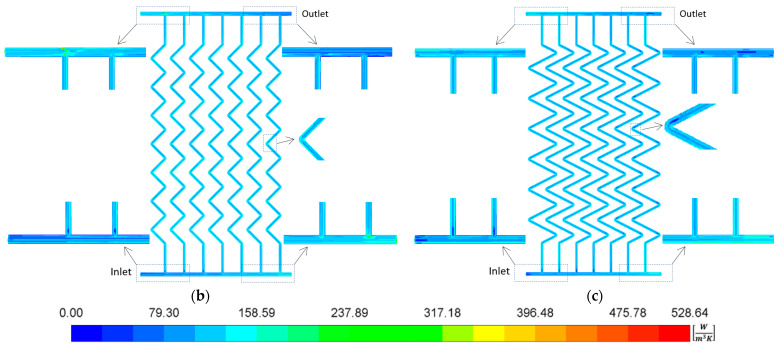
Local entropy generation rate due to heat transfer inside the FPCs at 3.0 L/min: (**a**) conventional, (**b**) zigzag type A, and (**c**) zigzag type B.

**Figure 13:**
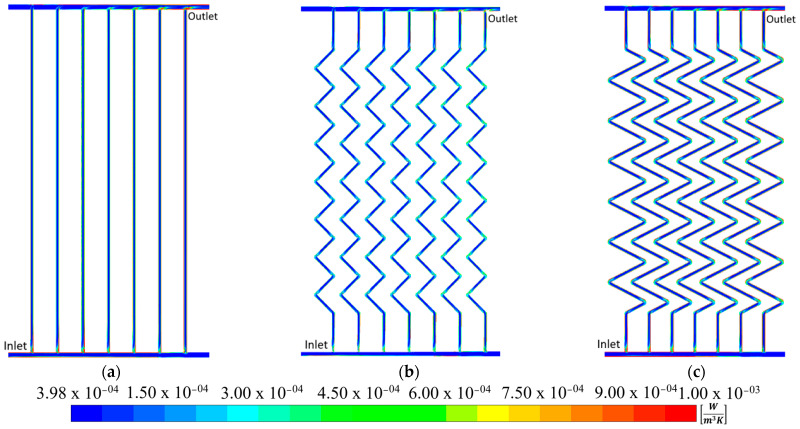
Local entropy generation rate due to fluid viscosity inside the FPCs at 3.0 L/min: (**a**) conventional, (**b**) zigzag type A, and (**c**) zigzag type B.

The local *S_h_* and *S_µ_* in the interior of the pipes for the conventional (case 1), zigzag type A (case 2), and zigzag type B (case 3) configurations of the FPCs, considering a volumetric flow rate of 3.0 L/min, are illustrated in Figure 12 and Figure 13. It can be seen that the local *S_h_* throughout the lower and upper headers and the pipes of the FPCs (Figure 12) was related to the high temperature difference between the water and the surface of the pipe (Figure 5). Therefore, the zones with high entropy generation rates are related to the zones where the temperature gradients are high (Figure 5). For example, the conventional geometry exhibited the highest temperature gradients in the lower and upper headers, opposite the inlet and the outlet of the fluid, respectively. Consequently, these zones showed the highest values in the entropy generation rate due to heat transfer (Figure 5 and Figure 12).

The higher *S_µ_* for the three FPCs occurred near the walls of the tubes, in the sections where the fluid flow of the lower header was divided and distributed in the seven tubes of the FPCs, and in the section where the seven tubes fed the upper header of the FPCs (Figure 13). Finally, a higher entropy generation was observed in the zigzag geometries, specifically in cases 2 and 3, in the areas where the zigzag was formed. These behaviors were related to the velocity gradients that were inside the tubes of the FPCs (Figure 7). However, as discussed previously, this contribution of the *S_µ_* was insignificant.

## 4. Conclusions

A detailed performance comparison between conventional and zigzag tube geometries of flat plate solar collectors (FPCs) was conducted using Computational Fluid Dynamics (CFD) to analyze the entropy generation rates. Complete geometries in three dimensions and a variation of the inlet volumetric flow rates (from 1.0 to 9.0 L/min) were analyzed.

The zigzag geometry types A and B could obtain higher outlet temperatures than conventional geometry for all the volumetric flow rates. However, it is most convenient to use the zigzag type B geometry due to its highest outlet temperature. The maximum temperature difference was approximately of 7.5 K between the zigzag type B geometry and the conventional case at the lowest volumetric flow rate (1.0 L/min).

The pressure inside the flat plate solar collectors tended to increase in the three cases, while the volumetric flow rates increased after 2.0 L/min and were higher in the zigzag geometries. The highest difference in the maximum pressure between the zigzag type B and the conventional case was approximately 75%, and was reached at 9.0 L/min.

The maximum contribution of the entropy generation rate due to heat transfer with respect to the total entropy was approximately 4.2% for the zigzag type B geometry with a volumetric flow rate of 3.0 L/min. The entropy generation due to fluid viscosity for the operating condition considered for the three geometries was negligible. Therefore, the most important loss in the FPCs was due to the entropy generation rate due to heat loss.

Finally, it is worth mentioning that the results described above are valid only for the use of water as a working fluid due to the climatic conditions that prevail in Guanajuato, Mexico. To avoid fluid freezing problems in solar collectors, many countries work with glycol solutions, which will be considered in future work.

## Figures and Tables

**Table 1:** Parameters of the FPC and water properties.

Description	Specification
Number of tubes, [-]	7
External diameter, [m]	0.0254
Internal diameter, [m]	0.025
Width of the FPC, [m]	1
Length of the FPC, [m]	1.75
Area of the FPC, [m^2^]	1.75
Horizontal inclination of the FPC, [°]	21
Density, [kg∙m^−3^]	Boussinesq, Equation (7)
Thermal expansion coefficient, [K^−1^]	0.000206
Specific heat, [J∙kg^−1^∙K^−1^]	4182
Thermal conductivity, [W/m∙K]	0.6
Viscosity, kg∙m^−1^∙s^−1^	001003

**Table 2:** Mesh sensibility case 1, conventional geometry.

Number of Elements	Average Temperature [K]	Variation between the Previous Value of Average Temperature [K]	Convergence Time [Hour]
30,253	306.50	-	0.58
82,341	307.22	0.72	1.5
278,864	307.71	0.49	5.5
649,887	308.07	0.36	8.2
1,734,153	308.06	0.01	17.5

**Table 3:** Mesh sensibility case 2, zigzag type A geometry.

Number of Elements	Average Temperature [K]	Variation between the Previous Value of Average Temperature [K]	Convergence Time [Hour]
516,833	308.90	-	7.5
1,260,857	309.85	0.95	13.8
2,501,458	309.81	0.04	22.6

**Table 4:** Mesh sensibility case 3, zigzag type B geometry.

Number of Elements	Average Temperature [K]	Variation between the Previous Value of Average Temperature [K]	Convergence Time [Hour]
405,458	310.50	-	6.7
906,544	311.84	1.34	11.0
1,923,992	312.63	0.79	21.2
4,589,410	312.52	0.11	39.8

## Data Availability

Not applicable.
